# Artificial intelligence-assisted training for proximal caries diagnosis by undergraduate dental students: a randomized experimental study

**DOI:** 10.1186/s12909-026-09982-2

**Published:** 2026-07-22

**Authors:** Rana Alkattan, Lulwah Alreshaid, Hayam Alfallaj, Arun Gopinathan Pillai, Hussam M. Alqahtani, Omar Suhaym, Sami Almohefer, Ahmed A. Madfa

**Affiliations:** 1 Department of Restorative and Prosthetic Dental Sciences, College of Dentistry, King Saud bin Abdulaziz University for Health Sciences (KSAU-HS), Riyadh, 11426 Saudi Arabia; 2 King Abdullah International Medical Research Center, Ministry of National Guard Health Affairs, Riyadh, Saudi Arabia; 3 Dental Services, King Abdulaziz Medical City, Ministry of the National Guard- Health Affairs, Riyadh, Saudi Arabia; 4 Department of Maxillofacial Surgery and Diagnostic Sciences, College of Dentistry, King Saud bin Abdulaziz University for Health Sciences (KSAU-HS), Riyadh, Saudi Arabia; 5 Department of Preventive Dental Sciences, College of Dentistry, King Saud bin Abdulaziz University for Health Sciences (KSAU-HS), Riyadh, Saudi Arabia; 6 Department of Restorative Dental Science, College of Dentistry, University of Ha’il, Ha’il, Kingdom of Saudi Arabia

**Keywords:** Artificial intelligence, Dental education, Dental caries, Radiographs, Caries detection, Bitewings

## Abstract

**Background:**

Artificial intelligence (AI) has shown promise in improving the detection of proximal caries on dental radiographs; however, its educational value in enhancing undergraduate students’ independent radiographic interpretation remains unclear. Therefore, this study aimed to evaluate the effect of AI–assisted radiology training on undergraduate dental students’ diagnostic competence in assessing proximal caries extension when compared with conventional radiology training.

**Methods:**

This randomized experimental study included 82 undergraduate dental students. After completing a pre-test using 20 conventional bitewing radiographs, from which students evaluated three proximal surfaces per radiograph, students were randomly allocated to either conventional radiology training or AI-assisted training using Second Opinion^®^ software. Both groups received a one-hour training session. A post-test using 20 conventional bitewing radiographs without AI assistance was conducted one day later, with students again evaluating three proximal surfaces per radiograph. Diagnostic performance was assessed using total exact-match scores (0–60) and absolute average error (0–5 scale). Completion time in minutes was also recorded for pre- and post-tests. Mixed-design ANOVA was used to evaluate group, time, and interaction effects (α = 0.05).

**Results:**

Seventy-nine students completed both tests, with 41 trained using AI assistance and 38 trained using conventional radiography. No significant differences were found between training groups or across time points (pre- and post-intervention, *p* > 0.05). However, absolute average errors increased significantly in the conventional group from pre- to post-test (*p* < 0.05), indicating a greater deviation from the correct answers. Post-test completion time was significantly shorter in the AI group compared with the conventional group, and both groups demonstrated significant reductions in completion time from pre- to post-test (*p* < 0.05).

**Conclusions:**

AI-assisted training did not significantly improve students’ diagnostic competency in proximal caries detection compared with conventional radiology training. However, AI training improved diagnostic efficiency, as indicated by the lower absolute average errors, and did not impair students’ independent radiographic interpretation when AI support was removed. AI may serve as a supportive adjunct in undergraduate dental education, particularly for improving efficiency and consistency in lesion depth estimation.

## Introduction

Dental caries is considered the most prevalent disease worldwide and its management remains a significant challenge in clinical practice [1]. Given its high prevalence and progressive nature, the success of non-invasive management strategies relies heavily on early detection of lesions before structural breakdown occurs [2]. Initial caries lesions may develop asymptomatically and are often detected only incidentally on radiographic examination or after cavitation has already occurred [3]. Hence, for minimally invasive treatment options to be viable, lesions should be detected as early as possible. Once the caries lesion reaches beyond the dentino-enamel junction, the lesion is likely cavitated, and surgical treatment is typically recommended [4].

For proximal caries, bitewing radiographs are the most reliable method for detection, as visual-tactile examination may not always be feasible, specifically in early lesions with minimal to no clinical signs [5]. Because proximal surfaces are sometimes unavailable to direct inspection, bitewing radiography is thought to be the most accurate technique for identifying and evaluating proximal carious lesions. However, radiographic interpretation is linked with low diagnostic accuracy, especially for early enamel lesions, and is prone to significant variation across examiners [6]. Caries lesion detection using bitewing radiography has limited validity and reliability; significant disagreements are commonly observed between examiners, with a high proportion of false positive and false negative reports [7]. A systematic review on the caries detection of early and advanced carious lesions determined that nearly 76% of lesions were missed, indicating a low sensitivity. Specificity is somewhat greater, with false positives detected much less often [6]. Unsurprisingly, more experienced examiners were nearly four times better at correctly diagnosing proximal lesions than those with less experience [8]. These difficulties underscore the significance of successful teaching strategies that can enhance students’ radiographic interpretation abilities and promote the growth of diagnostic competence.

Artificial intelligence (AI) has recently become a popular tool in the analysis of dental radiographic images. In several recent studies, the use of AI significantly enhanced dentists’ diagnostic accuracy in the detection of proximal caries, with a marked improvement in the sensitivity for identifying early enamel lesions [9–12]. Recent developments in AI, especially deep learning-based image processing, have generated a lot of interest in dental radiography, as these AI-based systems can identify proximal caries on bitewing radiographs with diagnostic accuracy that is on par with or higher than that of dental professionals. Additionally, it has been demonstrated that AI-assisted interpretation increases physicians’ sensitivity for detecting carious lesions and may lower variability in radiographic evaluations [9]. Similar results were reported for diagnosis of dental alveolar lesions on periapical radiographs [13, 14]. As a result, the feasibility of integrating AI software into educational contexts has become more and more popular, with results demonstrating the viability of AI as an alternative to expert labeling of radiographic findings [15].

The accurate detection of proximal lesions is crucial in clinical decision-making and is an integral component of undergraduate dental education [12]. Thus, the educational application of AI in dentistry has emerged as a rapidly growing area of research. AI may be used as an instructional tool in addition to a diagnostic tool by offering visual help, making pattern identification easier, and assisting students as they learn. In one aspect, students who received AI-assisted training showed substantially greater caries detection accuracy than students who got traditional instructions alone [15], while another study showed that after receiving AI-supported instructions, students’ capacity to evaluate proximal enamel caries did not significantly increase [16]. These contradictory results imply that more research is necessary to determine the educational advantages of AI-assisted radiology training.

To date, the integration of commercially available AI-based software systems into undergraduate dental education has not been thoroughly investigated. Furthermore, it remains unclear whether incorporating AI-based software—particularly systems that rely on color-coded visualization for caries detection—would improve students’ diagnostic performance when AI assistance is no longer available. Most previous studies have focused on students’ performance while AI assistance is available during radiographic interpretation [12, 15]. Nevertheless, there is still insufficient data to determine if exposure to AI-assisted training enhances one’s capacity for independent diagnosis once AI help is eliminated. In undergraduate dental education, where students must acquire independent diagnostic skills applicable to actual clinical settings, this is very crucial. Hence, by comparing students’ diagnostic outcomes with and without prior AI-assisted training, the effect of such training on their ability to assess caries extension can be more clearly determined.

Therefore, the primary objective of this study was to evaluate the diagnostic competence of undergraduate dental students after radiology training, comparing students who received AI-assisted training with those who underwent conventional training without AI support. The primary outcome measures included diagnostic scores assessed against the consensus diagnoses established by experienced dentists. The secondary objective was to determine whether AI-assisted training would improve the speed at which students diagnose dental caries, measured as the time taken to complete the tests. The null hypotheses were as follows: (a) there will be no significant difference in the scores of caries extension diagnosis between students trained with AI and those without, (b) there will be no significant difference in the time required to complete diagnoses between students who received AI training and those who did not.

## Materials and methods

### Study setting

This randomized experimental study was conducted in the College of Dentistry, King Saud bin Abdulaziz University for Health Sciences (KSAU-HS), Riyadh, Saudi Arabia, and the College of Dentistry, University of Hail, Hail city, Saudi Arabia. Ethical approval was obtained from King Abdullah International Medical Research Center (KAIMRC) Institutional Review Board (NRR24/004/11).

### Participant recruitment

A power analysis using Cohen’s d of 0.596 was conducted to determine the required sample size. This analysis indicated that a sample size of 78 students would be necessary to detect a medium effect size with a power of 80% at a significance level of α = 0.05.

**Fig. 1:**
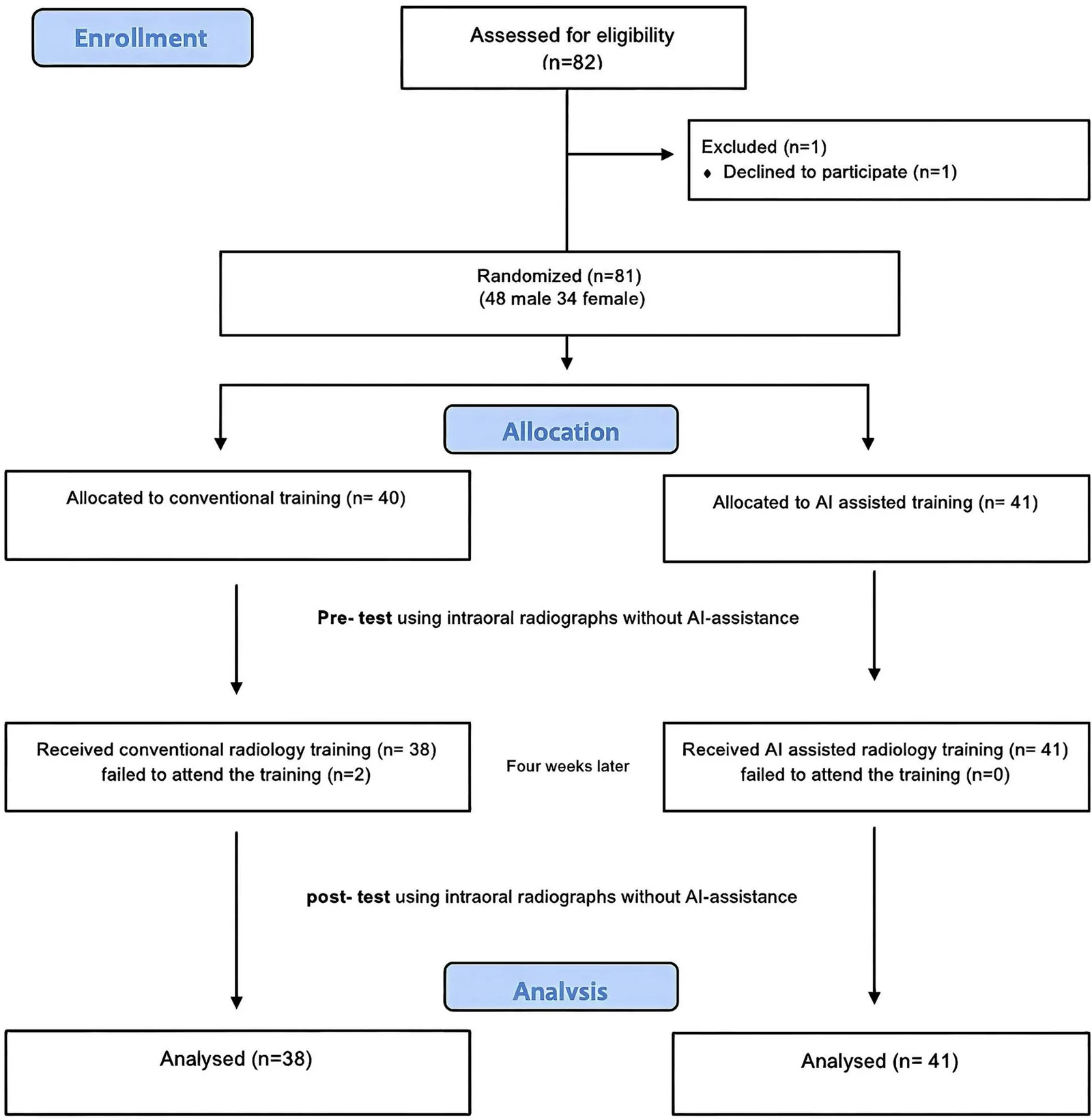
CONSORT flow diagram of the study

A total of 82 undergraduate dental students were enrolled in this study. The study was conducted at two dental schools to achieve the sample size required by the a priori power analysis. The study was designed with the educational objective of evaluating the educational carryover effect of AI-assisted training on students’ independent radiographic interpretation skills beyond conventional radiology training. The study was conducted at the end of the students’ pre-clinical year. Inclusion criteria included successful completion of the required radiology courses at their respective institutions and provision of written informed consent. The participating institutions followed comparable pre-clinical radiology curricula covering the principles of intraoral radiographic interpretation and dental caries diagnosis. Students from both institutions were also included in the same randomization process. The study was designed and reported in accordance with the Consolidated Standards of Reporting Trials (CONSORT) statement guidelines (Fig. 1) [17].

### Radiograph selection

A total of 60 intraoral bitewing radiographs were randomly selected from existing radiographic datasets available at KSAU-HS university. Only bitewings radiographs of permanent posterior teeth with fully visible dental crowns and no proximal overlap were included. Radiographs of teeth with proximal restorations, secondary caries, or third molars were excluded.

All radiographic images were evaluated in a dimly lit room using a consistent screen (MacBook Air, Apple, Cupertino, CA, USA). A panel of six experts, composed of restorative dentists with more than 10 years of clinical experience, independently reviewed the intraoral bitewing radiographic images to evaluate the depth of the proximal carious lesions. Classification was made using the proximal caries classification system with E1-E2, D1-D3 ratings [18]. The ratings were coded following an ordinal scale: 0 = no caries/sound, 1 = superficial enamel caries limited to the outer half of the enamel (E1), 2 = deep enamel caries extending to the inner half of the enamel (E2), 3 = initial dentin caries limited to the outer third of the dentine (D1), 4 = moderate dentin caries extending to the middle third of the dentine (D2), 5 = deep dentin caries extending to the inner third of the dentine (D3). Following independent evaluations, radiographs were reviewed collectively by the expert panel. The final reference standard was established through expert consensus among the six evaluators to determine the key answers. During these consensus discussions, the AI software (Second Opinion^®^, Pearl AI, Mountain View, CA, USA) was consulted only for radiographs in which disagreement existed among the experts. The software did not assign E1–D3 diagnostic classifications; rather, it provided visual annotations outlining suspected carious lesions. These annotations served only as supplementary information to facilitate discussion of lesion extent and boundaries, particularly for lesions identified by the software as “present with high confidence”. According to the software developer (Pearl Inc.), this setting restricts outputs to findings that meet the software’s internal confidence criteria; however, the exact numerical threshold for this classification is not publicly disclosed. The final lesion classification was determined solely by expert consensus, independent of the AI software.

### Study design

#### Pre-test

Upon consent, students completed a pre-test using conventional intraoral bitewing radiographs to assess their ability to determine caries extension. The pre-test consisted of 20 bitewing radiographs, from which students were asked to evaluate three proximal surfaces per radiograph. The assessment was conducted using iPad devices (Apple Inc., Cupertino, CA, USA). The time required to complete the test was recorded in minutes using a standardized timer. All answers were anonymous, and no identifying data were obtained from the participating students.

#### Training

The students were then randomly assigned a unique number. Afterwards, a computer-generated random number generator randomly assigned each student to one of two groups. The randomization sequence was generated by an investigator who was not involved in outcome assessment. Group assignments were concealed using sequentially numbered opaque envelopes until allocation was completed. Participants were assigned to groups by a separate investigator after completion of baseline procedures. Due to the nature of the educational intervention, participant blinding was not feasible. However, statistical analysis of outcomes was performed using anonymized participant codes to minimize assessment bias.

- Group 1: students received a one-hour training session on diagnosing dental caries using conventional intraoral radiographs without AI assistance. The session, conducted 4 weeks after the pre-test by a single trained restorative dentistry faculty member, included 20 bitewing radiographs not included in the pre-test.
- Group 2: students received a one-hour training session on diagnosing dental caries using intraoral radiographs with AI assistance using the AI software Second Opinion^®^. During the training, each radiograph was presented twice simultaneously: once with AI-generated color-coded diagnoses and once without AI. The software Second Opinion^®^ is an AI–based dental radiographic analysis software designed to assist in the detection of radiographic findings, including proximal caries. The software analyzes radiographic images and provides visual annotations highlighting areas suspected of containing carious lesions. During the training, students were presented with radiographs with AI-generated annotations, which included color-coded visual indicators designed to assist in identifying and localizing suspected proximal carious lesions. The software was used exclusively as an educational aid during the training session and was not available during the post-test assessment. The session used the same radiographs as Group 1 (20 bitewings) and was conducted 4 weeks after the pre-test by the same trained restorative dentistry faculty. No additional formal instruction related to proximal caries diagnosis was provided to either group during the four-week interval.

#### Post-test

**Fig. 2:**
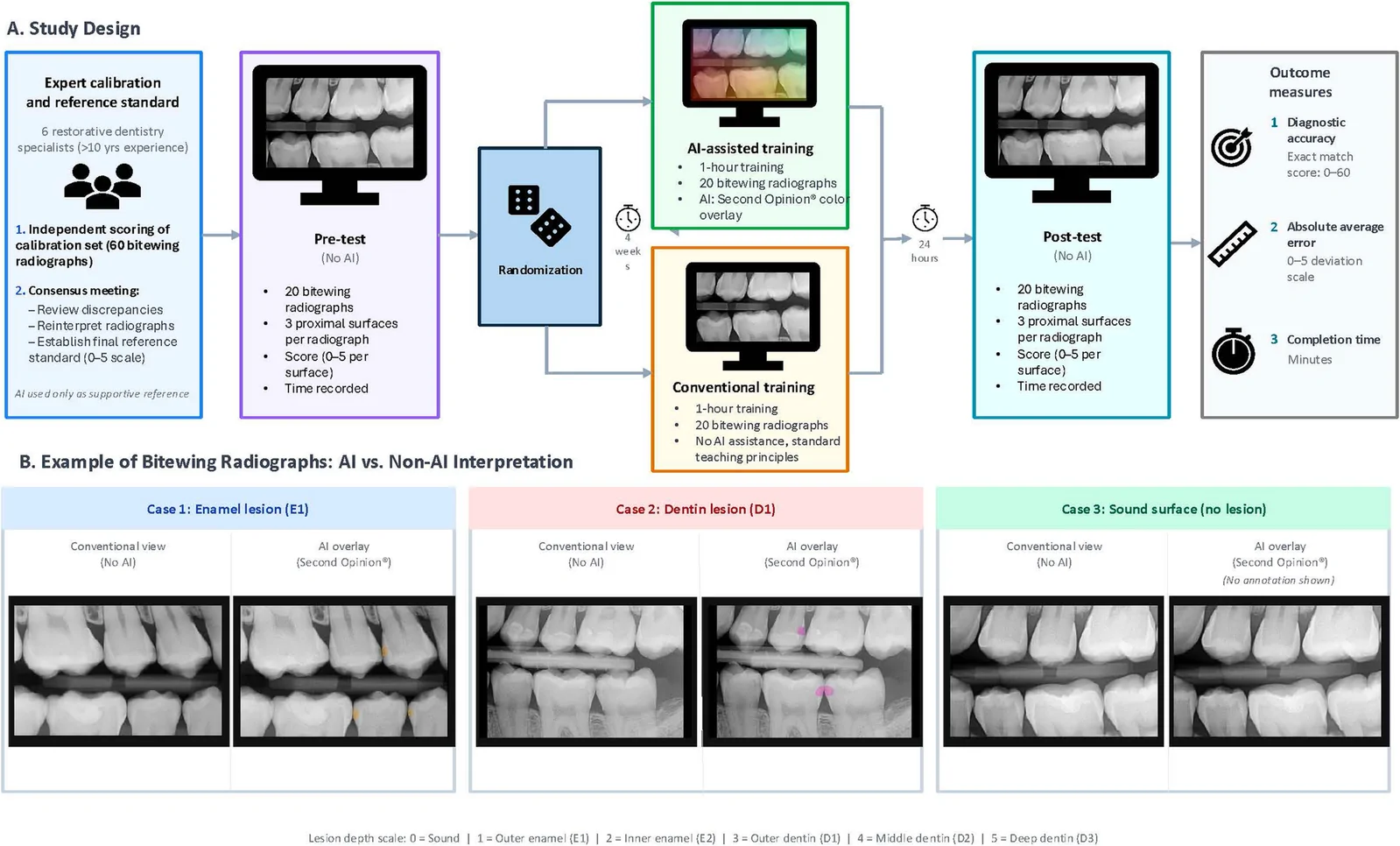
Schematic illustration of the study design and experimental workflow. **A**. Study design and **B**. Example of bitewing radiographs: AI vs non-AI interpretation

One day after the training, both groups underwent a post-test using conventional intraoral radiographs without AI assistance. The post-test consisted of 20 bitewing radiographs, none of which were used in the pre-test or training sessions, from which students were asked to evaluate three proximal surfaces per radiograph. Similar to the pre-test, the assessment was conducted on iPad devices (Apple Inc., Cupertino, CA, USA), and the time required to complete the test was recorded in minutes using a standardized timer. The use of iPads was chosen to reflect the standard educational setting in which students routinely perform radiographic interpretation and learning activities. A schematic illustration of the study design is shown in Fig. 2.

### Statistical analysis

Statistical analyses were conducted to compare the AI-assisted training group with the conventional training group in their pre- and post-intervention radiographic caries-detection performance. For secondary outcomes (absolute average error and completion time), no a priori power calculation was performed. As these outcomes were not the primary focus of the study, their analyses were considered exploratory. Consequently, statistically significant findings for these outcomes should be interpreted as hypothesis-generating rather than confirmatory. No adjustments for multiple comparisons were applied, as the secondary outcomes addressed distinct constructs and were not intended to form a single analytical family. For each participant and assessment, the total score was based on exact matches only, assigning one point for correct answers and zero points otherwise. The points were totaled to a score ranging from 0 to 60. The absolute average error was then used to calculate an absolute difference between the numeric student response and the numerical code assigned to the reference standard for each question, followed by averaging the errors across all questions. Each error value ranged from 0 (correct answer) to 5 (maximum possible score across the six categories of caries lesion classification). In other words, an error value of 0 indicated an exact match, whereas higher values reflected progressively greater deviations from the correct classification.

Group similarity at baseline was assessed using chi-square tests for categorical variables and independent-samples t-tests for continuous variables. The primary efficacy analyses used mixed-design ANOVA with one within-subjects factor (time: pre vs. post) and one between-subjects factor (group: AI vs. conventional) for each outcome, providing tests of the main effect of time, the main effect of group at post-test via simple main effects, and the time × group interaction to evaluate differential change between groups. Data were analyzed by IBM SPSS Statistics for Windows, Version 29 (IBM Corp., Armonk, NY, USA), and a level of significance of 0.05 was used for all inferential analyses, with *p*-values < 0.05 considered statistically significant.

## Results

### Inter-rater agreement

Considering the ratings as ordinal, inter-rater agreement between the six raters was evaluated using Krippendorff’s Alpha. Inter-rater agreement was calculated based on the independent evaluations before consensus discussions. An overall excellent level of agreement was found for all 120 lesions included in the pre- and post-tests (Krippendorff’s Alpha 0.805, 95% CI 0.778–0.833), indicating high consistency among raters and sufficient validity of the bitewing radiographic images for knowledge testing.

The six raters differed slightly in accuracy when their ratings were compared with the consensus (AI-determined key answer). Agreement ranged between 62.8% and 86.7% with exact match and between 90.3% and 100% with ± 1 unit of error allowed (difference in one category, for example, D1 vs. D2).

### Student enrollment

Of the 82 students eligible for enrollment in this study, 79 completed both pre- and post-test assessments, for a response rate of 96.3%. Gender distribution showed no significant difference among the groups (32 females and 47 males; χ² (1) = 0.03, *p* = 0.857). Of these students, 41 were trained using AI assistance while 38 were trained using conventional radiography.

### Diagnostic assessment

To confirm that the pre- and post-test assessments were comparable in difficulty, we compared the distribution of the consensus-derived correct answer categories (0 = sound to 5 = deep dentin caries) between the two test sets. Fisher’s exact test showed no significant difference in the frequency distribution of lesion categories (*p* = 0.194), and the Mann-Whitney U test indicated no significant difference in the ordinal severity of lesions between tests (*p* = 0.825). These findings support that the complexity and composition of the pre- and post-test radiograph sets were similar, allowing direct comparison of student performance over time.

**Fig. 3:**
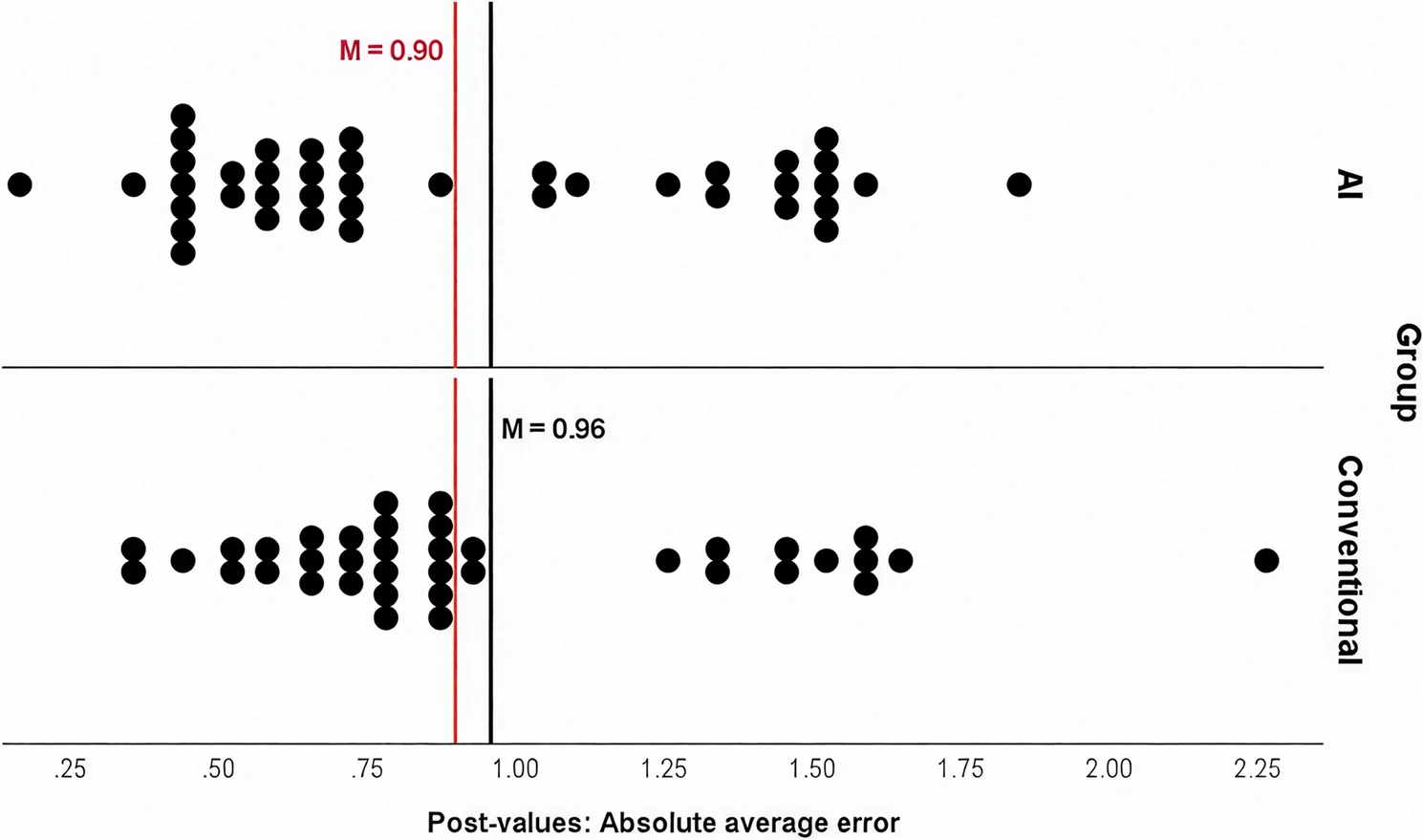
Dot plot showing absolute average error for post-test scores in AI-assisted and conventional groups. Group means are represented by the vertical lines

Table 1 shows that there was no significant difference between the AI-assisted and conventional groups in pre-test and post-test scores. Each group also showed similar assessment scores across time points (pre- and post-intervention), with no significant difference (Table 2). Absolute average errors increased significantly in the conventional group from pre- to post-test assessment (*p* = 0.025, Fig. 3), indicating an increase in their average deviation from the correct answers. 

**Table 1:** Descriptive statistics for pre- and post-test outcomes

Outcome	Group	*n*	Pre-test	Post-test	Change (Post - Pre)
Student scores (0 to 60)	AI	41	30.00 ± 10.60	27.71 ± 11.97	-2.29 ± 9.54
	Conv	38	28.76 ± 9.43	27.37 ± 10.47	-1.39 ± 8.69
Absolute average error (0 to 5)	AI	41	0.81 ± 0.44	0.90 ± 0.45	0.09 ± 0.36
	Conv	38	0.84 ± 0.39	0.96 ± 0.43	0.12 ± 0.31
Completion time (mins)	AI	41	17.15 ± 6.39	12.78 ± 5.45	-4.37 ± 5.59
	Conv	38	19.76 ± 7.81	15.76 ± 5.30	-4.00 ± 7.53

*Conv*  Conventional group, reported values are Mean ± SD

**Table 2:** Inferential statistics comparing groups and time points

Outcome	Comparison	*p*-value*	Cohen’s d	95% CI
Student scores (0 to 60)	Pre-test: AI vs. Conv^b^	0.586	0.12	-3.27, 5.75
	Post-test: AI vs. Conv^b^	0.894	0.03	-4.72, 5.40
	Time × Group interaction^c^	0.665	0.10	-5.01, 3.21
	Pre vs. Post: AI group^a^	0.131	0.24	-5.30, 0.71
	Pre vs. Post: Conv group^a^	0.333	0.16	-4.27, 1.49
Absolute average error (0 to 5)	Pre-test: AI vs. Conv^b^	0.744	0.07	-0.22, 0.16
	Post-test: AI vs. Conv^b^	0.517	0.15	-0.26, 0.13
	Time × Group interaction^c^	0.658	0.10	-0.18, 0.12
	Pre vs. Post: AI group^a^	0.121	0.25	-0.02, 0.20
	Pre vs. Post: Conv group^a^	**0.025**	0.38	0.02, 0.22
Completion time (mins)	Pre-test: AI vs. Conv^b^	0.106	0.37	-5.80, 0.57
	Post-test: AI vs. Conv^b^	**0.016**	0.55	-0.57, -5.40
	Time × Group interaction^c^	0.806	0.06	-3.32, 2.59
	Pre vs. Post: AI group^a^	**< 0.001**	0.78	-6.13, -2.60
	Pre vs. Post: Conv group^a^	**< 0.001**	0.53	-6.48, -1.52

*Conv*  Conventional group, *CI*  Confidence interval

^a^paired t-test comparing pre- vs. post-test within each group; ^b^between-group comparison (independent samples t-test); ^c^interaction term from mixed-design ANOVA

*p-value<0.05 is considered statistically significant

Regarding completion time, no significant differences were found between study groups in the pre-test; however, in the post-test, completion time was significantly shorter in the AI group than in the conventional group (*p* = 0.016). Completion time decreased significantly in both groups from pre- to post- test (Tables 1 and 2).

## Discussion

The present study is among the first to examine the impact of AI integration on dental students’ diagnostic performance during radiology training. While previous studies have shown that AI can significantly assist practicing dentists in accurately diagnosing proximal caries [10, 19, 20], our study aimed to determine whether dental students’ ability to diagnose the extent of proximal caries would be influenced by the availability of AI software during their radiology training. An important aspect of the present study design is that AI assistance was available only during the training intervention and not during the post-test assessment. Consequently, the study evaluated the educational carryover effect of AI-assisted training rather than the diagnostic performance of students while actively using AI. This distinction is important because AI systems may improve diagnostic performance when available during image interpretation; however, the present findings specifically reflect whether exposure to AI-assisted learning influences students’ subsequent independent radiographic interpretation. Therefore, the results should not be interpreted as evidence regarding the effectiveness of real-time AI-supported diagnosis.

The findings revealed no significant differences in diagnostic scores for caries extension between students who received AI assistance and those who conducted assessments without AI training. Hence, the first null hypothesis was not rejected, suggesting that both training methods produced comparable levels of competency. The similar distribution of caries lesion categories across the assessments also suggests that test complexity and composition did not influence the observed outcomes. Consequently, these results indicate that AI integration did not significantly enhance overall diagnostic competency when compared to conventional radiology training methods.

A slight decrease in diagnostic scores from pre- to post-test was observed in both study groups, although these differences were not statistically significant. A likely explanation is that all participants had already completed their formal pre-clinical radiology training before enrollment. Consequently, the intervention was introduced after students had already acquired the foundational knowledge and skills required for radiographic interpretation, making it unlikely that a single one-hour educational session would produce measurable improvements beyond their existing level of competency. Nonetheless, several other factors should also be considered. Radiographic interpretation of proximal caries, particularly early enamel lesions, remains diagnostically challenging due to subtle radiographic changes and inherent observer variability [7, 21]. In addition, the intervention consisted of a relatively brief single-session training exercise, which may have been insufficient to produce measurable short-term improvements in diagnostic accuracy. Repeated assessment of multiple radiographic images may also have contributed to cognitive fatigue or increased uncertainty during lesion classification. Importantly, despite the slight reduction in scores, students demonstrated relatively low absolute error values, suggesting that overall performance remained stable and clinically acceptable.

When comparing the current study’s findings with those of previously published studies, Ayan et al. reported significantly higher post-test caries detection accuracy among students trained with AI software than among those who were not [15]. This difference may be related to variations in study design: both groups initially lacked prior dental radiology training, with only one group receiving AI-based instructions. In contrast, in the current study, no statistically significant difference was observed between conventional and AI-assisted training, as both groups had previously completed their course-based radiology training, after which they were exposed to a standardized educational intervention. While the AI-trained group was exposed to color-coded lesion visualization, the conventional training group relied on standard radiographic interpretation without such visual aids. Similarly, Schropp et al. assessed the impact of AI software use on students’ ability to detect enamel caries [16]. The study found no statistically significant difference in detection ability between the test group that received AI-assisted training and the control group that received no AI training. Nonetheless, some differences in study design should also be noted. In Schropp et al.’s study, the participating students already had previous clinical experience, and the software version used was limited to identifying the presence or absence of enamel caries without assessing lesion extent. Moreover, caries identification was shown to be hampered by proximal tooth overlap.

Furthermore, in the current study, the time required to complete the post-test was significantly shorter than that for the pre-test, indicating an improvement in diagnostic efficiency rather than diagnostic competency. Additionally, students trained using AI completed the post-test significantly faster than those who received conventional training; hence, the second null hypothesis was rejected. This difference may be associated with the characteristics of the AI training, which included exposure to radiographs both with and without color-coded lesion visualization, potentially facilitating more efficient radiographic interpretation during the assessment [22]. A recent study by Arsiwala-Scheppach et al. found that the availability of AI during radiographic interpretation lead to more efficient viewing behavior and less time taken to notice caries compared to the absence of AI [23]. In other words, dentists utilizing AI fixated longer on teeth with carious lesions and/or restorations compared to sound teeth. However, AI assistance was not available during the post-test in the present investigation. Therefore, the observed reduction in completion time among students who received AI-assisted training may reflect a potential educational carryover effect rather than the direct influence of real-time AI support. In contrast, Ayan et al. reported that students who received AI training required a significantly longer time to complete their post-test, which may be attributed to the absence of prior dental radiology training [15].

Several methodological strengths reinforce the validity of the present findings. The use of a randomized experimental design allowed for the assessment of AI integration while controlling for potential confounding factors. Additionally, the inclusion of students from two different institutions across two cities allows the findings to be more broadly applicable. Finally, the strong consensus among six experts in evaluating intraoral bitewing radiographic images ensured a reliable assessment of proximal carious lesion depth, thereby improving the accuracy and consistency of the reference standard. Previous studies have shown that factors such as clinical experience can influence the reliability of dental radiographic interpretation [24], further supporting the importance of an expert-based reference standard.

The high inter-rater agreement among the six expert evaluators indicates strong consistency in lesion classification and supports the reliability of the diagnostic benchmark. The validity of the assessment is an important aspect of the present study, as it confirms the robustness of the reference standard. In addition, the absence of significant differences in the distribution of caries lesion categories between the pre- and post-tests confirms that both assessments were comparable in complexity. The use of experienced clinicians to establish the reference standard is particularly relevant, as diagnostic accuracy in radiographic interpretation has been shown to improve with clinical experience [8, 24]. Therefore, comparing students’ performance against expert consensus provides a meaningful framework for evaluating their diagnostic competence.

Radiographic diagnosis of dental caries is particularly challenging for early enamel lesions due to their subtle grey-scale variations, which often result in low contrast on radiographs [21]. Consequently, conventional radiography has been reported to demonstrate low to moderate diagnostic accuracy, with sensitivities ranging from 24% to 42%, particularly for initial enamel caries lesions [6, 25]. Despite these challenges, the present study found that the absolute average error values were consistently below 1, indicating that students generally estimated the extent of caries lesions with reasonable accuracy and minimal deviation from the reference standard. This suggests that, although exact classification of lesion depth may be difficult, students were typically able to provide clinically acceptable estimations following both AI-assisted and conventional training. This finding is clinically relevant because the radiographic estimation of lesion depth plays an important role in treatment planning and decision-making. In particular, differentiating between lesions confined to enamel and those extending into dentin may influence preventive, non-invasive, or restorative management strategies. Therefore, the relatively small deviations observed from the expert reference standard suggest that both educational approaches enabled students to make lesion-depth assessments that were generally aligned with clinically meaningful diagnostic thresholds, even when exact agreement with the reference standard was not achieved. From a clinical perspective, it is also important to recognize that radiographic findings do not always precisely correspond to the true clinical extent of caries lesions [4, 6]. The interpretation of proximal caries is inherently associated with a degree of uncertainty, particularly in early enamel lesions where radiographic changes are subtle [6, 7]. Therefore, small deviations in classification, such as within one category, may fall within an acceptable diagnostic range and may not necessarily represent clinically significant errors [2, 4].

Regarding secondary outcomes, the conventional training group demonstrated a significant increase in absolute average error from pre- to post-test, whereas no such increase was observed in the AI-assisted group, even though overall diagnostic scores were comparable between both groups. This exploratory pattern may indicate a difference in the stability of lesion depth estimation between the groups. However, because this outcome was not pre-specified as primary and no correction for multiple comparisons was applied, this result should be interpreted with caution and considered hypothesis-generating rather than definitive. One possible explanation is that AI-generated visual cues provide a standardized reference for lesion localization and extent during training, which may reduce subjective interpretation and promote more uniform evaluation strategies. This observation is consistent with previous studies indicating that AI applications can reduce subjectivity and interobserver variability in dental radiographic interpretation and improve consistency in diagnostic assessments [26–28].

It is also important to consider the participants’ level of experience. The included students were at an early stage of their dental education, and their diagnostic skills were still developing. Radiographic interpretation requires repeated exposure and experience to achieve proficiency [8, 24]. In contrast, the reference standard in this study was established by experienced clinicians with greater diagnostic expertise. This difference in baseline competency may have limited the extent to which a short AI-assisted training session could influence performance, as students may not yet have developed the foundational skills required to fully benefit from AI-supported learning. Nonetheless, the findings of the present study support the increasing incorporation of AI into dental education, particularly for the radiographic diagnosis of dental caries [9, 12]. Despite the growing interest in AI-assisted learning, concerns remain that reliance on such tools may limit students’ ability to independently interpret conventional radiographs and accurately diagnose caries in the absence of AI support. In the present study, however, students who received AI-assisted training demonstrated diagnostic performance comparable to that of students trained using conventional methods, with no statistically significant differences in diagnostic accuracy observed between the two groups. These findings suggest that exposure to AI, including color-coded lesion visualization, did not negatively affect students’ independent diagnostic abilities when AI support was removed.

While AI-assisted training did not result in improved diagnostic accuracy, it may still serve as a supportive educational tool. The use of color coding may facilitate visual recognition, guide attention to relevant areas of radiographic images, and support pattern identification, particularly for early learners. In this context, AI may help reduce cognitive load during the learning process, even if this does not immediately translate into measurable improvements in performance [23]. Therefore, AI should be considered a complementary tool that supports, rather than replaces, conventional teaching methods [12, 15]. However, these findings should be interpreted with caution, and further research is required to determine the long-term educational impact and generalizability of AI integration across different learning environments.

This study has several limitations. First, the power calculation was designed specifically for the primary outcome of diagnostic scores. Secondary outcomes, specifically absolute average error and completion time, were not powered a priori, and, as such, findings for these measures should be interpreted cautiously. Second, the training intervention consisted of a single one-hour session, which may not be sufficient to fully demonstrate the potential impact of AI-assisted learning. Furthermore, all participants had previously completed their formal pre-clinical radiology training before enrollment. As a result, the intervention was introduced after students had already acquired foundational radiographic interpretation skills, potentially limiting the magnitude of any additional educational benefit attributable to the intervention. Longer and repeated exposure to AI tools, particularly when introduced earlier in the learning process, may be necessary to observe meaningful improvements in diagnostic performance. Third, the study was conducted in a pre-clinical setting and did not evaluate the application of AI in real clinical scenarios. The selected radiographs did not include diverse datasets and hence may not fully reflect the variability and complexity encountered in routine clinical practice. Finally, while all participants had successfully completed the required pre-clinical radiology courses before enrollment, potential differences in institutional teaching approaches, students’ prior experience, or familiarity with radiographic interpretation were not fully controlled and may have influenced the outcomes. A formal comparison of the radiology curricula between the participating institutions was not undertaken. Future research integrating both theoretical training and clinical application is necessary to better understand how AI tools influence diagnostic performance in real-world settings.

## Conclusion

The current study demonstrated that integrating AI into undergraduate dental radiology training did not significantly improve students’ diagnostic competency in assessing proximal caries compared with conventional training methods. Although AI-assisted training was associated with improved diagnostic efficiency, no significant differences in overall diagnostic accuracy or lesion depth estimation were observed between groups. The absence of measurable improvements may, in part, reflect the limited duration of the AI-based intervention and the participants’ prior completion of formal pre-clinical radiology training. Furthermore, the AI-assisted group did not demonstrate the increase in absolute average error observed in the conventional training group. As this was an exploratory secondary outcome, it should be interpreted cautiously as hypothesis-generating and warrants confirmation in future studies.

Importantly, exposure to AI-based lesion coding did not compromise students’ ability to independently interpret radiographs when AI support was removed. However, the findings reflect the educational carryover effect of AI-assisted training rather than the impact of real-time AI-assisted diagnosis. These findings suggest that AI can be incorporated into dental education as a supportive adjunct to conventional teaching approaches, rather than a replacement. While short-term exposure to AI may not enhance diagnostic accuracy, it may facilitate learning by improving efficiency and supporting visual interpretation. Future research is needed to evaluate the long-term impact of AI-assisted training and its integration into clinical education and practice.

## Data Availability

The datasets used and/or analysed during the current study are available from the corresponding author on reasonable request.
